# Fever Unmasked Brugada Syndrome in Pediatric Patient: A Case Report

**DOI:** 10.5811/cpcem.2020.2.44418

**Published:** 2020-04-23

**Authors:** Orhay Mirzapolos, Perry Marshall, April Brill

**Affiliations:** Midwestern University, Department of Emergency Medicine, Downers Grove, Illinois

**Keywords:** Brugada, pediatric, viral illness, arrhythmias, fever

## Abstract

**Introduction:**

Brugada syndrome is an arrhythmogenic disorder that is a known cause of sudden cardiac death. It is characterized by a pattern of ST segment elevation in the precordial leads on an electrocardiogram (EKG) due to a sodium channelopathy.

**Case Report:**

This case report highlights the case of a five-year-old female who presented to the emergency department with a febrile viral illness and had an EKG consistent with Brugada syndrome.

**Discussion:**

Fever is known to accentuate or unmask EKG changes associated with Brugada due to temperature sensitivity of the sodium channels.

**Conclusion:**

Febrile patients with Brugada are at particular risk for fatal ventricular arrhythmias and fevers should be treated aggressively by the emergency medicine provider. Emergency medicine providers should also consider admitting febrile patients with Brugada syndrome who do not have an automatic implantable cardioverter-defibrillator for cardiac monitoring.

## INTRODUCTION

Brugada syndrome is an important cause of sudden cardiac death and is prevalent in a young patient population. Nevertheless, the classic electrocardiogram (EKG) findings associated with the disease may not always be present in individuals with Brugada syndrome.^1^ The characteristic ST-segment elevations in the precordial leads are caused by a loss of function mutation of sodium channels involved in phase 0 of the cardiac cycle.^2^ A new hypothesis is that these sodium channels are also temperature sensitive. As a result, a fever accentuates impaired sodium channels influx to not only unmask classic Brugada EKG changes, but may also induce potentially fatal ventricular arrhythmias.^1^ As demonstrated in this case report, a young female presented to the emergency department (ED) for the evaluation of a febrile viral illness and was found to have this life-threatening cardiac anomaly on her EKG.

## CASE REPORT

A five-year-old African-American female presented to a community hospital ED with two days of subjective fevers. This fever was associated with nasal congestion, a productive cough, a sore throat, and injected conjunctiva. She also complained of nausea, and her mother reported decreased oral intake. Her mother denied any history of syncopal episodes. The patient was born full term and had no past medical history. Her immunizations were up to date with the exception of an annual influenza vaccine. Furthermore, she had no notable family medical history and, specifically, no family history of sudden cardiac death.

In the ED she was ill appearing, but she was well-hydrated and non-toxic in appearance. Her vitals were as follows: temperature 101 degrees Fahrenheit, heart rate 118 beats per minute, blood pressure 105/70 millimeters of mercury, respiratory rate 20 breaths per minute, saturating 98% on room air.

On physical exam, her ears, nose and throat exam were only notable for congestion and an erythematous pharynx. Her lungs were clear to auscultation bilaterally. On cardiac exam, she was tachycardic with an irregular rhythm. There were no murmurs, rubs, or gallops heard. Her abdomen was soft and non-tender. Lastly, her skin exam was unremarkable.

Her complete blood count (CBC), complete metabolic panel (CMP), magnesium, and troponin were within normal limits, but did test positive for influenza A. Her chest radiograph was negative. Her EKG (Image) showed a sinus rhythm with frequent premature ventricular contractions, a right axis, and coved ST elevation in V1–V2.

Upon recognition of the Brugada pattern on her EKG, it was determined that she required higher level of care than was available at the community hospital. She was transferred to a children’s hospital and evaluated by pediatric cardiology. While there, an echocardiogram was performed and was negative for structural heart disease. She also underwent an electrophysiology study, which was negative for inducible arrhythmias. Thus, the decision was made to defer automatic implantable cardioverter-defibrillator (AICD) placement. The patient and her family were referred for genetic testing, but they chose not to complete the testing. Currently, she is followed regularly by a pediatric cardiologist and has had no adverse cardiac events to date.

**CPC-EM Capsule**

What do we already know about this clinical entity?

Brugada syndrome is an important cause of sudden cardiac death as a result of a loss of function mutation of sodium channels.

What makes this presentation of disease reportable?

Fever provokes electrocardiogram (ECG) changes in asymptomatic individuals with Brugada and the ECG may revert to normal when the patient becomes afebrile.

What is the major learning point?

The impaired sodium channels are thought to be temperature sensitive. Fever is the state most likely to induce ventricular arrhythmia in children with Brugada.

How might this improve emergency medicine practice?

Physicians must be cognizant of the potentially life-threatening complications of fevers in this patient population and have a low threshold for admission.

## DISCUSSION

Brugada syndrome was first recognized in 1992 and has since been identified as an autosomal dominant, inherited sodium channelopathy.^3^ Specifically, a loss of function mutation of the SCN5A gene causes an impairment of sodium influx during phase 0 of the cardiac cycle.^2^ Classic EKG findings include three types of ST elevations in V1–V3: coved, saddleback, or a combination.^3^ The diagnostic criteria for Brugada syndrome is the presence of a type I pattern (coved ST elevation) on EKG with one of the following: documented ventricular arrhythmia; family history of sudden cardiac death; type I pattern EKG in a family member; induced ventricular arrhythmia with electrical stimulation; or history of unexplained syncope.^4^

Fever provokes these EKG changes in asymptomatic individuals with Brugada and the EKG may revert to normal when the patient becomes afebrile. While the exact mechanism is unknown, it is postulated that the sodium channels are temperature sensitive and thus fever exacerbates the impairment of sodium influx through the channel.^1^ This leads to nonhomogeneous repolarization of the right ventricle and thus creates a potential for re-entry arrhythmias.^3^ In fact, fever has been associated with 18% of cardiac arrests in patients with Brugada.^4^ In children, symptomatic Brugada is most frequently associated with fever.^7^ As clinicians, it is important to recognize that fever is the state most likely to induce ventricular arrhythmia in children with Brugada.^5^ Parents need to be educated to treat fevers aggressively and hospital admission for cardiac monitoring should be considered for patients without an AICD. Furthermore, EPs should not be falsely reassured by the normalization of the EKG in this patient population.^6^

Treatment of asymptomatic patients with Brugada is controversial in adults and not well studied in pediatrics. Symptomatic patients with a history of arrhythmia, syncope, or family history of sudden cardiac death should all receive an AICD. Those who are asymptomatic commonly undergo electrophysiology studies and if no inducible arrhythmia is found, they are typically managed conservatively with close follow-up.^3^

## CONCLUSION

Brugada syndrome is primarily reported in adults, with limited data on its presentation in pediatrics. In both adults and pediatrics, a febrile illness may unmask an underlying Brugada pattern EKG, even in individuals with previously normal EKGs. The impaired sodium channels are thought to be temperature sensitive, and thus fever exaggerates the impaired sodium influx. This not only emphasizes the EKG changes, but also places the patient at risk for fatal arrhythmias. This case report demonstrates an instance in which these EKG changes were only revealed as a result of a febrile illness. While there have been a handful of similar case reports published in the literature, it is still a novel concept for many clinicians. EPs in particular must be cognizant of the potentially life-threatening complications of fevers in this patient population and have a low threshold for admission.

## Figures and Tables

**Image:**
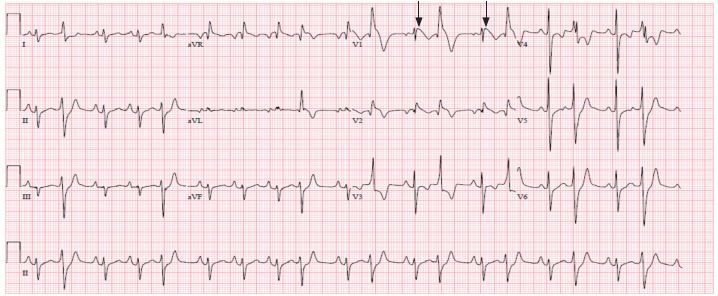
Patient’s electrocardiogram showing Brugada pattern coved ST elevation in V1–V2 (arrows).
